# Carotenoids from Foods of Plant, Animal and Marine Origin: An Efficient HPLC-DAD Separation Method

**DOI:** 10.3390/foods1010052

**Published:** 2012-12-19

**Authors:** Irini F. Strati, Vassilia J. Sinanoglou, Lintita Kora, Sofia Miniadis-Meimaroglou, Vassiliki Oreopoulou

**Affiliations:** 1 Laboratory of Food Chemistry and Technology, School of Chemical Engineering, National Technical University of Athens, Iroon Polytechniou 5, Zografou 15780, Athens, Greece; E-Mail: vasor@chemeng.ntua.gr; 2 Instrumental Food Analysis Laboratory, Department of Food Technology, Technological Educational Institution of Athens, Ag. Spyridonos 12210 Egaleo, Greece; E-Mail: v_sinanoglou@yahoo.gr; 3 Food Chemistry Laboratory, Department of Chemistry, University of Athens, Panepistimioupolis Zographou 15701, Athens, Greece; E-Mails: kora@yahoo.gr (L.K.); miniadou@chem.uoa.gr (S.M.-M.)

**Keywords:** carotenoids, HPLC, photodiode array detection, tomato waste, *Penaeus kerathurus*, avian egg yolks

## Abstract

Carotenoids are important antioxidant compounds, present in many foods of plant, animal and marine origin. The aim of the present study was to describe the carotenoid composition of tomato waste, prawn muscle and cephalothorax and avian (duck and goose) egg yolks through the use of a modified gradient elution HPLC method with a C_30_ reversed-phase column for the efficient separation and analysis of carotenoids and their *cis*-isomers. Elution time was reduced from 60 to 45 min without affecting the separation efficiency. All-*trans* lycopene predominated in tomato waste, followed by all-*trans*-β-carotene, 13-*cis*-lutein and all-*trans* lutein, while minor amounts of 9-*cis*-lutein, 13-*cis*-β-carotene and 9-*cis*-β-carotene were also detected. Considering the above findings, tomato waste is confirmed to be an excellent source of recovering carotenoids, especially all-*trans* lycopene, for commercial use. Xanthophylls were the major carotenoids of avian egg yolks, all-*trans* lutein and all-*trans* zeaxanthin in duck and goose egg yolk, respectively. In the *Penaeus kerathurus* prawn, several carotenoids (zeaxanthin, all-*trans*-lutein, canthaxanthin, cryptoxanthin, optical and geometrical astaxanthin isomers) were identified in considerable amounts by the same method. A major advantage of this HPLC method was the efficient separation of carotenoids and their *cis*-isomers, originating from a wide range of matrices.

## 1. Introduction

Carotenoids, a group of more than 600 naturally occurring fat-soluble pigments, have attracted many researchers, because of their commercially desirable properties, such as their natural origin, null toxicity and high versatility. They also provide both lipo- and hydro-soluble colorants and provitamin A [1]. Carotenoids can be synthesized by plants, algae, yeast, fungi and photosynthetic bacteria and contain 40 carbon atoms. They are classified into carotenes (e.g., β-carotene, α-carotene, lycopene) and xanthophylls (e.g., β-cryptoxanthin, lutein, zeaxanthin, canthaxanthin). These compounds show antioxidant and immunomodulation activities, which may prevent degenerative diseases, such as cardiovascular diseases, diabetes and several types of cancer, especially prostate and digestive-tract tumors [2]. For instance, both lycopene and β-carotene may exhibit a cholesterol synthesis inhibiting effect that may enhance low density lipoprotein degradation. In addition, oxygenated carotenoids, such as lutein, have been shown to be associated with a reduced risk of cardiovascular disease [3]. Of the various carotenoids in plants, lycopene has been reported to exhibit the highest antioxidant activity, followed by β-cryptoxanthin, β-carotene, lutein and zeaxanthin [4].

Tomato is a food product containing large amounts of carotenoids, mainly in the form of lycopene and β-carotene. Its waste, generated as a by-product during tomato processing in large quantities, is considered as an important source of natural carotenoids, and therefore, attention has been given to the determination of its carotenoid profile. On the other hand, egg yolks contain two xanthophylls with important health benefits (lutein and zeaxanthin), which have higher bioavailability than those from plant sources, probably because the lipid matrix of the egg yolk facilitates greater absorption [5]. Additionally, the predominant carotenoid of shrimp, astaxanthin, presents more than ten-fold higher antioxidant activity than zeaxanthin, lutein, canthaxanthin and β-carotene and more than one-hundred-fold than α-tocopherol [6].

High-performance liquid chromatography (HPLC) has been employed as a powerful technique to quantify low levels and various forms of carotenoids in foods. The analysis of carotenoids has been routinely performed by reversed-phase HPLC, because of its improved separation efficiency [7]. C_30_ columns provide better resolutions between carotenoids with similar polarity compared to C_18_ columns, so they are normally the columns of choice for the separation of geometrical isomers [8,9].

The objective of the present study was to apply a modified HPLC method with a C_30_ reversed-phase column for the efficient separation and analysis of carotenoids and their *cis*-isomers. An attempt has also been made to reduce the elution time without affecting the separation efficiency. The ultimate goal was to provide a method suitable to identify and quantify all the carotenoid isomers present in plant (tomato waste), marine (*Penaeus kerathurus* prawn) and animal (egg yolk from avian species) sources.

## 2. Experimental Section

### 2.1. Reagents and Standards

All the solvents used for sample preparation and extraction were of analytical grade and were obtained from Merck (Darmstadt, Germany). All solvents used for HPLC analysis (acetonitrile, 1-butanol and methylene chloride) were of HPLC grade and were obtained from Merck (Darmstadt, Germany). All-*trans* carotenoid standards (lycopene, β-carotene, lutein, zeaxanthin, canthaxanthin and astaxanthin) were purchased from Sigma Chemical Co. (Sigma-Aldrich Company, St. Louis, MO, USA).

### 2.2. Instrumentation

The HPLC (Hewlett Packard Series 1100, Waldbronn, Germany) system was composed of a HP 1100 Quaternary Pump, an Agilent 1100 Series Micro Vacuum Degasser, a Rheodyne model 7010 Sample Injector and a HP 1100 Series Diode Array Detector (DAD). The HPLC system was equipped with a YMC (Tokyo, Japan) C_30_ column (250 × 4.6 mm I.D., 5 μm particle). The analysis of the chromatographic data was carried out on a ChemStation for LC 3D software (Agilent Technologies, Waldbronn, Germany).

### 2.3. Extraction of Carotenoids from Various Matrices

#### 2.3.1. Tomato Waste

Tomato processing waste (500 kg), composed of skin and seeds, was collected from NOMIKOS (Aliartos, Boeotia, Greece), a tomato-processing factory. Moisture content was determined at fresh tomato processing waste upon arrival at the laboratory and was found to be 80.48% ± 0.35%. The moisture content was determined according to the AOAC method 925.09 (1999). Tomato pomace was air dried at 25 °C, upon arrival, and the dry material (moisture content = 7.65% ± 0.21%) was subsequently vacuum packed in plastic bags and stored at −20 °C. Prior to each extraction, 60 g of dried tomato waste was homogenized in a domestic blender and finally powdered in a laboratory mill (Type ZM1, Retsch GmbH, Haan, Germany) equipped with different particle size sieves. Dry ground material was kept in glass jars wrapped with aluminum foil at −20 °C.

Carotenoids were extracted according to the method described by Strati and Oreopoulou [10]. Homogenized and ground dry tomato waste samples (10.00 g) were stirred with 100 mL acetone (tomato waste/solvent = 1:10) in a 500 mL extraction vessel equipped with a vertical water cooler and multiple-neck lid. The vessel was placed in a temperature-controlled (±1 °C) water bath and continuously agitated with a propeller type stirrer. The extraction temperature and time were kept constant at 25 ± 1 °C and 30 min, respectively. The mixture was vacuum filtered (Filter funnel DURAN 25 852 34, SCHOTT DURAN, Mainz, Germany); the solid residue was collected and re-extracted two more times, with fresh extraction solvent under the same conditions. Extracts were combined and centrifuged at 3000 rpm (HERMLE centrifuge Z380, Gosheim, Germany) for 10 min to separate the supernatant. Then, the supernatant was evaporated to dryness in a rotary vacuum evaporator (Rotavapor RE 111, Flawil, Switzerland) at 32 °C, dissolved in 1 mL methylene chloride and transferred to a vial. To prevent oxidation, *t*-butyl-hydroquinone, at a concentration of 100 mg/L, was added to all samples. The new solution was filtered through a 0.45 μm membrane filter, and 20 μL were injected for HPLC analysis. All samples were analyzed within three days after extraction. The whole extraction procedure was performed under dimmed light.

#### 2.3.2. Foods of Animal (Duck and Goose Egg Yolks) and Marine (*Penaeus kerathurus* Prawn) Origin

Duck (*Anas platyrhucus*) and goose *(Anser anser)* conventional eggs, less than three days after laying, were obtained from a local producer in Lakonia, Greece (three sampling repetitions) [11]. Egg yolks were separated manually from their respective whites, then homogenized in a blender, vacuum-packed in plastic bags and stored at −20 °C. All samples were analyzed within three days after extraction. Before analysis, samples were allowed to achieve room temperature.

Fifty adult Caramote prawns (*Penaeus kerathurus*) were caught in the North Aegean Sea (near Platamona Bay) in October 2010. Prawns were brought to the laboratory alive and individually measured for weight and length (with an average of 28.5 g/prawn and 16.7 cm/prawn, respectively). Then samples of muscle and cephalothorax were collected, individually weighed and homogenized.

As carotenoids are lipid constituents, total lipids from the above samples (duck and goose egg yolks, as well as prawn muscle and cephalothorax) were extracted according to Bligh and Dyer [12]. After phase equilibration, the lower chloroform layer (total lipids) was removed and dried in a rotary vacuum evaporator (Rotavapor RE 111, Flawil, Switzerland) at 32 °C. The extracted lipids were redissolved in chloroform/methanol (9:1, by volume). To prevent oxidation, *t*-butyl-hydroquinone, at a concentration of 100 mg/L, was added to all samples. All samples were analyzed within three days after extraction. The whole extraction procedure was performed under dimmed light.

### 2.4. HPLC Analysis of Carotenoids

For the identification of the different carotenoids, tomato waste extract and total lipids from avian (duck and goose) egg yolks and* Penaeus kerathurus *muscle and cephalothorax were further analyzed by HPLC-photodiode array detection. Before being injected, lipid samples were dried under nitrogen gas and dissolved in acetone:hexane (2:3, by volume). Afterwards, samples were filtered through a 0.45 μm membrane filter to remove particulate residues. Twenty microliters of solution were injected for the HPLC analysis. The solvent systems selected were based on several previous studies [13,14] and preliminary experiments using carotenoid standards. The most appropriate solvent system was found to be composed of acetonitrile, 1-butanol and methylene chloride. A mobile phase of acetonitrile (A), 1-butanol (B) and methylene chloride (C) with the following gradient elution was used: 69.3% A, 29.7% B and 1.0% C, initially; increased to 67.2% A, 28.8% B and 4% C, in the first 10 min; 61.6% A, 26.4% B and 12% C, after 20 min; 49% A, 21% B and 30% C, after 40 min; and returned to 69.3% A, 29.7% B and 1% C, after 50 min. The UV-visible spectra were obtained between 250 and 600 nm. The flow rate was maintained at 2 mL/min and the column temperature at 25 °C. The separation efficiency was evaluated on the basis of capacity factor (*k*) and separation (selectivity) factor (*α*), as follows [15]:

*k *= (*t*_R_−*t*_0_)/*t*_0_ (1)

*α* = *k*_B_/*k*_A_ (2)

where *t*_R _is the retention time of the peak of interest, *t*_0_ is the unretained peak’s retention time and *k*_A_ and *k*_B _are the capacity factors for peaks A and B, respectively [15]. 

### 2.5. Identification and Quantification of Carotenoids

The identification of *trans* and *cis* isomers of carotenoids was carried out by comparing the retention times and absorption spectra with reference standards and absorption spectra characteristics, as described in the literature [13,14].

Quantification was performed based on absolute calibration curves of all-*trans* lutein (447 nm), all-*trans* zeaxanthin (453 nm), all-*trans* canthaxanthin (452 nm), all-*trans* astaxanthin (478 nm), all-*trans*-β-carotene (455 nm) and all-*trans*-lycopene (476 nm), with a minimum of five concentration levels. For all-*trans* lutein, two standard curves were prepared, due to the great difference in lutein content found in the different samples. The concentration range for the carotenoid standard curves were: 1–20 μg/mL (low concentration) and 20–200 μg/mL (high concentration) for all-*trans*-lutein, 20–200 μg/mL for all-*trans* zeaxanthin, 50–500 μg/mL for all-*trans* canthaxanthin, 500–2000 μg/mL for all-*trans* astaxanthin, 2–40 μg/mL for all-*trans*-β-carotene and 10–100 μg/mL for all-*trans*-lycopene. The above concentration ranges were chosen as the most appropriate for determination of individual carotenoid content and were based on preliminary experiments.

The *cis* isomers of carotenoids were quantified using the standard curves of the corresponding all-*trans* carotenoids, because of the similarity in extinction coefficient [16]. Neolutein and cryptoxanthin were quantified using the curve of all-*trans* lutein and astaxanthin isomers and esters using the curve of all-*trans* astaxanthin. The carotenoids of all samples were quantified on a dry weight basis.

### 2.6. Determination of Limits of Detection (LOD) and Limits of Quantification (LOQ)

The limits of detection (LOD) and limits of quantification (LOQ) were determined using the calibration curves according to the method described by ICH [17].

The LODs and the LOQs were calculated according to the Equations (1) and (2):

LOD = 3.3 × *σ*/*S* (3)

LOQ = 10 × *σ*/*S* (4)

where *S* is the mean of the slopes of calibration curves and *σ* the standard deviation of the response [17].

### 2.7. Statistical Analysis

Three independent samples were analyzed and values were averaged and reported along with the standard deviation (SD). All statistical calculations were performed with the SPSS package (IBM SPSS Statistics, version 19.0, Chicago, IL, USA) statistical software for Windows. 

## 3. Results and Discussion

Initially, the HPLC method developed by Lin and Chen was tested for the determination of carotenoids in processed tomato juice [7]. However, the analytical conditions did not show good separation efficiency (some overlapping peaks), and the analysis itself took very long (more than 60 min, data not shown). We, therefore, improved the analytical method in terms of solvent percentages of mobile phase in order to cope with the above problems. After several preliminary trials, a gradient mobile phase consisting of acetonitrile, 1-butanol and methylene chloride, as described in the method section, was applied for the analysis of carotenoids and their isomers from different carotenoid matrices. 

The correlation coefficients achieved were for all-*trans* lutein (*y* = 21.04*x* + 33.40, *R*^2^ = 0.99 for low concentration and *y* = 24.93*x* − 1.07, *R*^2^ = 0.98 for high concentration), for all-*trans* zeaxanthin (*y* = 15.02*x* + 51.21, *R*^2^ = 0.98), for all-*trans* canthaxanthin (*y* = 18.93*x* + 23.91, *R*^2^ = 0.98), for all-*trans* astaxanthin (*y* = 7222.2*x* + 2842.5, *R*^2^ = 0.98), for all-*trans*-β-carotene (*y* = 19.97*x* + 57.20, *R*^2^ = 0.98) and for all-*trans* lycopene (*y* = 132.87*x* − 18.09, *R*^2^ = 0.99), where *y* denotes peak area and *x* concentration (μg/mL). 

The LODs for all-*trans* lutein, zeaxanthin, canthaxanthin, astaxanthin, all-*trans*-β-carotene and all-*trans* lycopene were 0.47, 1.54, 2.09, 57.0, 1.32 and 2.86 μg/mL, respectively, while the LOQs were 1.42, 4.66, 6.34, 173.0, 4.0 and 8.6 μg/mL. 

### 3.1. HPLC Analysis of Carotenoids in Tomato Waste Extract

**Figure 1:**
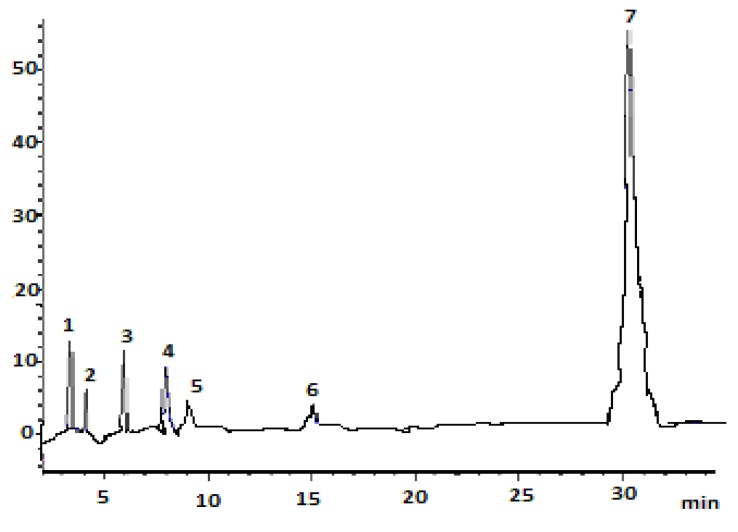
HPLC chromatogram of carotenoids in tomato waste acetone extract. The identified peaks include: (**1**) all-*trans*-lutein; (**2**) 9-*cis*-lutein; (**3**) 13-*cis*-lutein; (**4**) all-*trans*-β-carotene; (**5**) 9-*cis*-β-carotene; (**6**) 13-*cis*-β-carotene; and (**7**) all-*trans*-lycopene.

The HPLC chromatogram of carotenoids in tomato waste extract is presented in Figure 1. Seven carotenoids were separated and identified from the tomato waste acetone extract within 30 min. The chromatogram indicates that good separation efficiency and an adequate separation time were achieved for the analysis of carotenoids in tomato waste. Table 1 presents the chromatographic and the quantification data for the carotenoids in tomato waste. The *k* value (capacity factor) is used to assess the solvent strength of the mobile phase. The *k* values of all peaks ranged from 0.53 to 12.74, indicating that a proper solvent strength of the mobile phase was controlled. It has been reported that for optimum separation, the *k *values should range from 2 to 10, however, they can range between 0.5 and 20, when complicated compounds are to be separated [13]. The separation or selectivity factor (*α*) values for all the peaks were greater than 1.0, implying that a good selectivity of mobile phase to sample components was achieved.

**Table 1:** Tentative identification, chromatographic data and content (μg/100g dry basis) for all-*trans* and *cis* forms of carotenoids in tomato waste.

Peak No.	Compound	RT(min)	λ (nm)Found	λ (nm) Reported	*Q-*Ratio Found	*Q-*Ratio Reported	*k*	*α*	Content (μg/100 g Dry Basis )
				[7,13,14,16,18,19]		[7,13,14,16,18,19]			
1	All- *trans*-lutein	3.37 ± 0.04	423, 447, 477	422, 446, 476	0.04	0.06	0.53	2.06	39.14 ± 0.21
2	9- *cis-*lutein	4.94 ± 0.06	350, 420, 442, 474	356, 428, 446, 476	0.10	0.12	1.24	1.32	17.59 ± 0.05
3	13 *-cis-*lutein	5.91 ±0.09	376, 437, 458, 485	374, 434, 458, 488	0.31	0.33	1.69	1.51	42.69 ± 0.03
4	All- *trans*-β-carotene	7.97 ± 0.03	428, 454, 482	458, 482	-	0.12	2.62	1.15	48.48 ± 0.92
5	9 *-cis*-β-carotene	8.90 ± 0.08	340, 449, 480	344, 452, 476	0.11	0.12	3.04	1.88	4.24 ± 0.01
6	13- *cis*-β-carotene	15.09 ± 0.05	345, 451, 479	344, 422, 458, 476	0.34	0.35	5.86	2.15	4.42 ± 0.01
7	All- *trans*-lycopene	30.23 ± 0.20	450, 476, 507	452, 476, 506	-	0.06	12.74	2.15	64.84 ± 0.87

RT: retention time; *k*: capacity factor; *α*: separation (selectivity) factor; three independent samples were analyzed; data are expressed as mean ± standard deviation (*n* = 3).

Three all-*trans* carotenoids, namely lutein, β-carotene and lycopene, were identified based on criteria described in the experimental section. The visible absorption spectrum of peak 1 was 423, 447 and 477 nm (Table 1) and was identical to the all-*trans* lutein standard used in this study. Following the same approach, peaks 4 and 7 were identified as all-*trans*-β-carotene and all-*trans* lycopene, respectively. Peaks 2 and 3 were identified as 9-*cis* lutein and 13-*cis* lutein, respectively, because a low (at 350 nm) and a high (at 376 nm) intensity *cis* peak were observed. Additionally, a hypsochromic shift of 5 nm was observed for 9-*cis* lutein. It is reported that the mono-*cis *isomers of carotenoids result in a hypsochromic shift, when compared to the parent all-*trans* forms. Moreover, the presence of central isomers, such as 13-*cis* or 15-*cis*-carotenoids, would result in a significant absorption in the ultraviolet region (320–380 nm) [13]. Accordingly, and for the same reasons as above, peaks 5 and 6 were identified as 9-*cis-*β-carotene and 13-*cis*-β-carotene, respectively, because a hypsochromic shift of 5 and 3 nm occurred in the maximum absorption peak and two *cis *peaks (at 340 and 345 nm) appeared in the obtained spectra of the above mentioned peaks. The *Q*-ratio is defined as the ratio of height at *cis*-peak to the height at maximum absorption peak and may also be used to identify the *cis*-isomers [18]. As it can be observed in Table 1, all *cis-*isomers identified had *Q*-ratios very similar to those reported in the literature [13,14,16,18,19].

The carotenoid content of tomato waste (Table 1) was calculated based on the calibration curves of the respective standards, as described in the experimental section. All-*trans* lycopene predominated in tomato waste (64.84 ± 0.87 μg/100 g dry waste), followed by all-*trans*-β-carotene, 13-*cis*-lutein and all-*trans* lutein. Minor amounts of 9-*cis*-lutein, 13-*cis*-β-carotene and 9-*cis*-β-carotene were also detected (Table 1). Considering the above findings, tomato waste is confirmed to be an excellent source of recovering carotenoids, especially all-*trans* lycopene, for commercial use.

### 3.2. HPLC Analysis of Carotenoids in Avian (Duck and Goose) Egg Yolks

**Figure 2:**
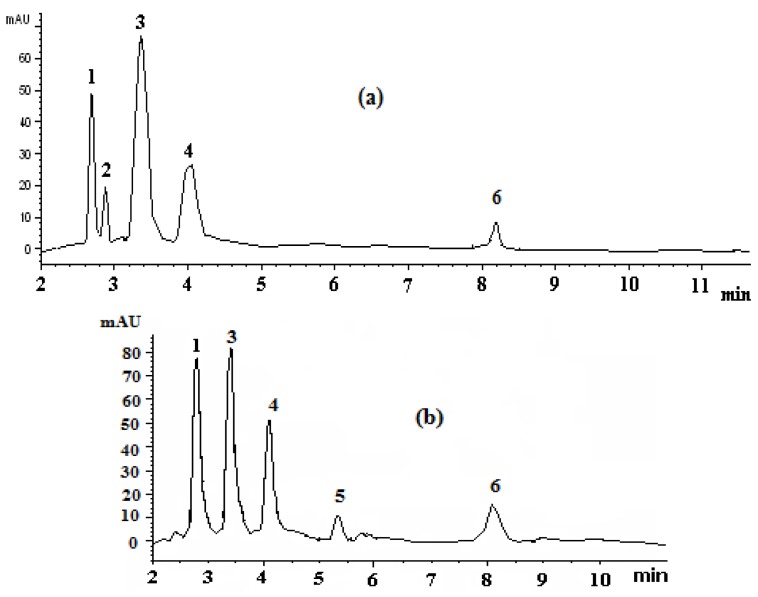
HPLC chromatograms of (**a**) duck and (**b**) goose egg yolk carotenoids. The identified peaks include: (**1**) all-*trans* zeaxanthin;(**2**) neolutein; (**3**) all-*trans *lutein; (**4**) all-*trans* canthaxanthin; (**5**) β-cryptoxanthin and (**6**) all-*trans*-β-carotene.

Six carotenoids were separated and identified from duck and goose egg yolks within 10 min. Figure 2 presents the chromatograms of duck (a) and goose (b) egg yolk carotenoids. Both chromatograms show good separation efficiency and a very short resolution time. Table 2 presents the identification data of these major egg yolk carotenoids, based on the chromatographic information obtained from the spectra. The *k* values of all peaks ranged from 0.60 to 2.83, indicating the adequacy of solvent strength of the mobile phase and its separation capacity (*α *values for all the peaks were greater than 1.0).

**Table 2:** Chromatographic identification and quantification data for duck and goose egg yolk carotenoids

Peak No.	Compound	RT(min)	λ (nm) found	λ (nm) reported	*Q*-Ratio Found	*Q*-Ratio Reported	*k*	*α*	Content (mg/100 g Wet Weight) [ 11]	
				[7,13,16,20,21,22]		[7,13,16,20,21,22]			Duck	Goose
1	All- *trans* zeaxanthin	2.75 ± 0.08	429, 450, 478	424, 454, 478	0.08	0.06	0.60	1.11	3.52 ± 0.80	6.22 ± 1.53
2	Neolutein ( *cis* isomer of lutein)	2.82 ± 0.02	330, 422, 443, 471	332, 442	0.24	-	0.63	1.77	1.50 ± 0.49	-
3	All- *trans *lutein	3.36 ± 0.05	425, 448, 476	426, 448, 472	0.08	0.06	0.89	1.57	9.88 ± 1.10	5.16 ± 0.61
4	All- *trans* canthaxanthin	4.07 ± 0.06	428, 454, 480	428, 452, 478	0.10	0.08	1.02	1.60	4.76 ± 3.41	3.84 ± 2.64
5	β-Cryptoxanthin	5.24 ± 0.02	428, 450, 477	428, 454, 480	0.17	0.16	1.47	1.92	-	1.17 ± 0.17 *
6	All- *trans*-β-carotene	8.12 ± 0.02	433, 455, 481	426, 454, 478	0.08	0.12	2.83	1.92	**	0.27 ± 0.02

RT: retention time; *k*: capacity factor; *α*: separation (selectivity) factor; three independent samples were analyzed; data are expressed as mean ± standard deviation (*n* = 3); * β-cryptoxanthin was quantified using the curve of all-*trans* lutein based on the similarity of its spectra characteristics with lutein; ** β-carotene amount was within the detection limits but lower than the quantification limits.

Peaks 1, 3, 4 and 6 were positively identified as all-*trans* zeaxanthin, all-*trans *lutein, all-*trans* canthaxanthin and all-*trans*-β-carotene, based on comparison of retention times and absorption spectra characteristics with those of the respective standard compounds used in this study. Additionally, the *Q*-ratios found were close to the ones reported in the literature [13,16,20,21]. The absorption maxima of peak 2 (identified as neolutein) showed a relatively small hypsochromic shift of 5 nm with respect toall-*trans*-lutein, which suggests that this is probably a *cis* isomer of lutein. The location of the *cis* double bond in this isomer is unknown; however, the presence of a strong *cis* peak in the UV region at 330 nm indicates that this *cis* double bond occupies a more central position, *i.e.*, 13-, 13′- or 15-, 15′-*cis* isomer [13]. Due to the absence of a commercial standard, peak 5 was tentatively identified as all-*trans*-β-cryptoxanthin, based on spectral characteristics and *Q*-ratios reported in the literature [22], and it was quantified using the curve of all-*trans* lutein based on the similarity of its spectra characteristics with lutein.

Quantitative data for avian egg yolk carotenoids are reported in the author’s previous study, and the respective data for duck and goose egg yolk carotenoids are tabulated in Table 2 [11]. According to this study, all-*trans* lutein was the predominant carotenoid in duck egg yolk (Figure 2a) (50.51% of total carotenoids), followed by all-*trans* canthaxanthin (24.36%), all-*trans* zeaxanthin (17.99%) and neolutein (7.65%). On the other hand, the prevailing carotenoid of goose egg yolk (Figure 2b) was all-*trans* zeaxanthin (37.32% of total carotenoids), followed by all-*trans* lutein (30.96%), all-*trans* canthaxanthin (23.05%), cryptoxanthin (7.04%) and all-*trans*-*β-*carotene (1.63%).

### 3.3. HPLC Analysis of Shrimp Carotenoids

**Figure 3:**
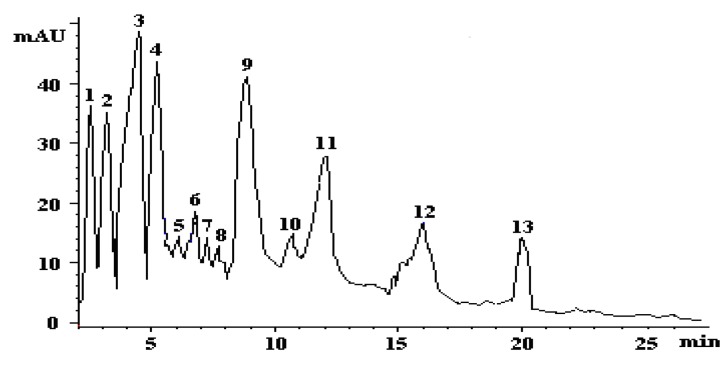
HPLC chromatogram of carotenoids in *Penaeus kerathurus* cephalothorax. The identified peaks include: (**1**) all-*trans* zeaxanthin; (**2**) all-*trans *lutein; (**3**) all-*trans* canthaxanthin; (**4**) β-cryptoxanthin; (**5**) α-cryptoxanthin; (**6**–**8**) unidentified astaxanthin *cis*-isomers; (**9**) (3*R*,3′*R*)-*trans*-astaxanthin; (**10**) unidentified astaxanthin *cis-*isomer; (**11**) (3*S*,3′*S*)-*trans*-astaxanthin and (**12**,**13**) unidentified astaxanthin esters.

Crustaceans are known to contain various carotenoids, which are responsible for their characteristic colors and are considered as one of the important sources of natural carotenoids. HPLC analysis of carotenoids from shrimp *Penaeus kerathurus* muscle and cephalothorax lipids revealed the presence of thirteen carotenoids, as presented in Figure 3. Details of the identification of peaks shown in Figure 3 as well as quantification data are tabulated in Table 3. 

**Table 3:** Chromatographic identification and quantification data of carotenoids from *Penaeus kerathurus* shrimp.

Peak No.	Compound	RT(min)	λ (nm) Found	λ (nm) Reported	*Q*-Ratio Found	*Q*-Ratio Reported	*k*	*α*	mg/100 g Cephalothorax	mg/100 g Muscle
				[20,21,23,24,25,26]		[20,21,23,24,25,26]				
1	All- *trans* zeaxanthin	2.53 ± 0.03	429, 450, 478	424, 454, 478	0.09	0.06	0.37	1.0	2.29 ± 0.06	0.52 ± 0.03
2	All- *trans *lutein	2.96 ± 0.06	425, 448, 476	426, 448, 472	0.08	0.06	0.60	1.63	2.65 ± 0.05	0.32 ± 0.02
3	All- *trans* canthaxanthin	4.55 ± 0.09	428, 454, 480	428, 452, 478	0.11	0.08	1.45	2.43	4.12 ± 0.07	0.73 ± 0.03
4	β-Cryptoxanthin	5.26 ± 0.03	428, 450, 477	425, 454, 478	0.04	0.05	1.83	1.26	0.37 ± 0.02 ^a^	-
5	α *-*Cryptoxanthin	5.95 ± 0.05	427, 447, 472	423, 446, 473	-	0.06	2.21	1.20		
6	Unidentified astaxanthin *cis*-isomer	6.74 ± 0.05	474	-	0.18	-	2.63	1.19	2.70 ± 0.05 ^b^	0.42 ± 0.02 ^b^
7	Unidentified astaxanthin *cis-*isomer	7.21 ± 0.07	475	-	0.11	-	2.89	1.10		
8	Unidentified astaxanthin *cis-*isomer	7.84 ± 0.04	475	-	0.07	-	3.23	1.12		
9	(3 *R*,3′*R*)-*trans*-astaxanthin	8.83 ± 0.09	478	477.6	-	-	3.76	1.16		
10	Unidentified astaxanthin *cis-*isomer	11.39 ± 0.06	474	-	0.11	-	5.14	1.37		
11	(3 *S*,3′*S*)-*trans*-astaxanthin	11.98 ± 0.03	479	478.8	-	-	5.46	1.06		
12	Unidentified astaxanthin ester	16.07 ± 0.07	481	482.5	-	-	7.67	1.40	0.80 ± 0.05	0.14 ± 0.01
13	Unidentified astaxanthin ester	20.05 ± 0.05	483	482.5	-	-	9.82	1.28	0.37 ± 0.03	0.07 ± 0.01

RT: retention time; *k*: capacity factor; *α*: separation (selectivity) factor; three independent samples were analyzed; data are expressed as mean ± standard deviation (*n* = 3); ^a^ the value 0.37 ± 0.02 is the sum for β-cryptoxanthin and α*-*cryptoxanthin, this value is located under the column mg/100 g cephalothorax; ^b^ the values 2.70 ± 0.05 and 0.42 ± 0.02 are the sum for unidentified astaxanthin *cis*-isomer + unidentified astaxanthin *cis-*isomer + unidentified astaxanthin *cis-*isomer + (3*R*,3′*R*)-*trans*-astaxanthin + unidentified astaxanthin *cis-*isomer + (3*S*,3′*S*)-*trans*-astaxanthin.

The *k *(capacity factor) and the *α* (separation factor) values of all peaks ranged from 0.37 to 11.17 and 1.0 to 1.14, indicating a good separation capacity of the mobile phase. By comparing UV spectra of peaks 1–3 with those obtained from the corresponding standards and *Q*-ratios with reference values in the literature [20,21,23], peaks 1–3 were identified as zeaxanthin, all-*trans*-lutein and canthaxanthin. Peaks 4 and 5 were identified as β- and α-cryptoxanthin, respectively, based on the similarity of spectral characteristics and *Q*-ratios with those reported in the literature [24,25]. Peaks 9 and 11 had a maximum absorption wavelength of 478 and 479 nm, respectively. By comparing UV spectra of peaks 9 and 11 with those obtained from the corresponding standard and absorption wavelength with reference values in the literature [26], peaks 9 and 11 were identified as the optical isomers (3*R*,3′*R*)-*trans*-astaxanthin and (3*S*,3′*S*)-*trans*-astaxanthin. Astaxanthin possesses two identical asymmetric carbon atoms at C-3 and C′-3, producing three optical isomers with all-*trans* configuration, the two enantiomers (3*S*,3′*S*) and (3*R*,3′*R*) and the *meso*-astaxanthin (3*S*,3′*R*/3*R*,3′*S*) [26]. Turujman [27] reported that synthetic astaxanthin standard consists of 25% of each enantiomer and 50% of the *meso* form [27]; in accordance with the chromatographic profile of the astaxanthin standard used in the present study. The means of identifying the astaxanthin isomers was through the spectral shift or the shift of the maximum absorption wavelength. In comparison to the (3*R*,3′*R*) and (3*S*,3′*S*)-all-*trans*-astaxanthin (λ_max_ of 478 and 479 nm, respectively), the four peaks (6–8 and 10 in Figure 3) showed a small hypsochromic effect of 3–4 nm and were possibly assigned as *cis*-astaxanthin isomers. Bjerkeng reported that astaxanthin consists of three chiral *R*/*S *isomers and 272 possible geometrical *cis*/*trans *isomers of which the quantitatively most important are 9-*cis*-, 13-*cis*- and 15-*cis*-isomer [28]. Finally, the fractions that elute later (peaks 12 and 13 in Figure 3) showed similar absorption spectra with *trans*-astaxanthin, indicating astaxanthin-derived compounds; possibly astaxanthin fatty acid esters.

The *k *(capacity factor) and the *α* (separation factor) values of all peaks ranged from 0.37 to 11.17 and 1.0 to 1.14, indicating a good separation capacity of the mobile phase. By comparing UV spectra of peaks 1–3 with those obtained from the corresponding standards and *Q*-ratios with reference values in the literature [20,21,23], peaks 1–3 were identified as zeaxanthin, all-*trans*-lutein and canthaxanthin. Peaks 4 and 5 were identified as β- and α-cryptoxanthin, respectively, based on the similarity of spectral characteristics and *Q*-ratios with those reported in the literature [24,25]. Peaks 9 and 11 had a maximum absorption wavelength of 478 and 479 nm, respectively. By comparing UV spectra of peaks 9 and 11 with those obtained from the corresponding standard and absorption wavelength with reference values in the literature [26], peaks 9 and 11 were identified as the optical isomers (3*R*,3′*R*)-*trans*-astaxanthin and (3*S*,3′*S*)-*trans*-astaxanthin. Astaxanthin possesses two identical asymmetric carbon atoms at C-3 and C′-3, producing three optical isomers with all-*trans* configuration, the two enantiomers (3*S*,3′*S*) and (3*R*,3′*R*) and the *meso*-astaxanthin (3*S*,3′*R*/3*R*,3′*S*) [26]. Turujman [27] reported that synthetic astaxanthin standard consists of 25% of each enantiomer and 50% of the *meso* form [27]; in accordance with the chromatographic profile of the astaxanthin standard used in the present study. The means of identifying the astaxanthin isomers was through the spectral shift or the shift of the maximum absorption wavelength. In comparison to the (3*R*,3′*R*) and (3*S*,3′*S*)-all-*trans*-astaxanthin (λ_max_ of 478 and 479 nm, respectively), the four peaks (6–8 and 10 in Figure 3) showed a small hypsochromic effect of 3–4 nm and were possibly assigned as *cis*-astaxanthin isomers. Bjerkeng reported that astaxanthin consists of three chiral *R*/*S *isomers and 272 possible geometrical *cis*/*trans *isomers of which the quantitatively most important are 9-*cis*-, 13-*cis*- and 15-*cis*-isomer [28]. Finally, the fractions that elute later (peaks 12 and 13 in Figure 3) showed similar absorption spectra with *trans*-astaxanthin, indicating astaxanthin-derived compounds; possibly astaxanthin fatty acid esters.

The comparison among the muscle and cephalothorax carotenoids showed that all-*trans* canthaxanthin predominated in both tissues (30.98% and 33.18% of total carotenoids, respectively), followed by all-*trans* zeaxanthin, all-*trans* astaxanthin and all-*trans* lutein in muscle (23.63%, 19.09% and 14.54% of total carotenoids, respectively) and all-*trans*-astaxanthin, all-*trans* lutein and all-*trans* zeaxanthin in cephalothorax (20.30%, 19.92% and 17.22% of total carotenoids, respectively). Furthermore, lesser amounts of unidentified astaxanthin esters were also determined in both tissues. Cryptoxanthin was identified only in the cephalothorax. On a weight basis, it was observed that the cephalothorax presented a higher total carotenoid content than muscle (13.30 ± 0.10 and 2.20 ± 0.07 mg/100 g wet tissue, respectively) (Table 3). Results showed that *Penaeus kerathurus* muscle and cephalothorax may be a good alternative of a carotenoid-rich food destined for human consumption. In accordance to our findings, Sachindra *et al.* [29] reported that the total carotenoid content of four species of shrimp (*Penaeus monodon*, *Penaeus indicus*, *Metapenaeus dobsonii *and *Parapenaeopsis stylifera*) from the Indian coast ranged from 10.4 to 17*.*4 ppm in the meat, from 35.8 to 153*.*1 ppm in the head and from 59.8 to 104*.*7 ppm in the carapace [29]. Howell and Matthews [30] found that farmed blue *P. monodon *shrimp exhibited low carotenoid concentrations (4.3–7.0 ppm) compared to those in wild shrimp (26.3 ppm) [30]. Carotenoid contents of shrimps vary, depending on their native habitat or manufactured diets [31].

## 4. Conclusions

A HPLC-DAD method was applied to separate the all-*trans*-carotenoids and their *cis*-isomers by employing a C_30_ column and a gradient mobile phase. A total of eight all-*trans* carotenoids, namely zeaxanthin, lutein, canthaxanthin, β-cryptoxanthin, α-cryptoxanthin β-carotene, astaxanthin and lycopene, as well as *cis*-isomers, were identified and quantified in representative carotenoid sources of plant (tomato waste), marine (*Penaeus kerathurus* prawn) and animal (egg yolk from avian species) origin. Major advantages of the method are: (1) a rapid and efficient separation and quantification of carotenoids; (2) high selectivity in the separation of all peaks of carotenes and xanthophylls, of all-*trans* and *cis*-isomers and of astaxanthin and its esters; and (3) the successful application in different matrices.
